# Immunomodulation of intracranial melanoma in response to blood-tumor barrier opening with focused ultrasound

**DOI:** 10.7150/thno.47983

**Published:** 2020-07-11

**Authors:** Colleen T. Curley, Aaron D. Stevens, Alexander S. Mathew, Katarzyna Stasiak, William J. Garrison, G. Wilson Miller, Natasha D. Sheybani, Victor H. Engelhard, Timothy N.J. Bullock, Richard J. Price

**Affiliations:** 1 Department of Biomedical Engineering, University of Virginia, Charlottesville, VA; 2 Department of Pathology, University of Virginia, Charlottesville, VA; 3 Carter Immunology Center and Department of Microbiology, Immunology, and Cancer Biology, Charlottesville, VA; 4 Department of Radiology & Medical Imaging, University of Virginia, Charlottesville, VA

**Keywords:** focused ultrasound, blood-tumor barrier, inflammation, immune cells, RNA sequencing

## Abstract

**Background:** Focused ultrasound (FUS) activation of microbubbles (MBs) for blood-brain (BBB) and blood-tumor barrier (BTB) opening permits targeted therapeutic delivery. While the effects of FUS+MBs mediated BBB opening have been investigated for normal brain tissue, no such studies exist for intracranial tumors. As this technology advances into clinical immunotherapy trials, it will be crucial to understand how FUS+MBs modulates the tumor immune microenvironment.

**Methods and Results:** Bulk RNA sequencing revealed that FUS+MBs BTB/BBB opening (1 MHz, 0.5 MPa peak-negative pressure) of intracranial B16F1cOVA tumors increases the expression of genes related to proinflammatory cytokine and chemokine signaling, pattern recognition receptor signaling, and antigen processing and presentation. Flow cytometry revealed increased maturation (i.e. CD86) of dendritic cells (DCs) in the meninges and altered antigen loading of DCs in both the tumor and meninges. For DCs in tumor draining lymph nodes, FUS+MBs had no effect on maturation and elicited only a trend towards increased presentation of tumor-derived peptide by MHC. Neither tumor endothelial cell adhesion molecule expression nor homing of activated T cells was affected by FUS+MBs.

**Conclusion:** FUS+MBs-mediated BTB/BBB opening elicits signatures of inflammation; however, the response is mild, transient, and unlikely to elicit a systemic response independent of administration of immune adjuvants.

## Introduction

Secondary brain tumors, arising from metastatic spread of extracranial malignancies, are the most common type of brain tumor 1. Up to 20% of cancer patients develop brain metastases, and this number could increase as treatments for primary disease extend patient survival 2. Malignant melanoma has one of the highest propensities for metastasizing to the brain 3. Following the development of brain metastases, patients are faced with a significantly worse prognosis and limited treatment options. Immune checkpoint inhibitors are a class of immunotherapeutic antibodies that have shown remarkable success in a subset of melanoma patients, and recent evidence has demonstrated some efficacy for these therapies in patients with metastatic brain tumors 4,5. However, it is possible that poor penetration of these therapies into the brain parenchyma and the unique immunological milieu of brain limit the efficacy of immunotherapy in the treatment of brain tumors.

A number of factors may contribute to poor penetration and limited efficacy of therapeutics in secondary brain tumors. Like primary brain tumors, metastatic brain tumors develop leaky vasculature. This results in high interstitial fluid pressure and limited convective transport of agents into the tissue, referred to as the blood-tumor barrier(BTB) 1. Despite regions of high vascular permeability, permeability varies between, and even within, single metastatic lesions 6. Additionally, delivery of therapeutic agents to brain metastases is significantly lower compared to peripheral metastases, possibly owing to retention of blood-brain barrier-like properties within a subset of vessels 1,6. Beyond challenges to effective therapeutic delivery, the brain has long been thought of as being relatively quiescent immunologically 7,8. Given these limitations, methods that may improve penetration and efficacy of immunotherapeutics in metastatic brain tumors are greatly needed.

Focused ultrasound applied in the presence of circulating microbubbles (MBs) has the ability to improve delivery and penetration of agents into brain tumor tissue, and therefore is an attractive modality for use in combination with traditional and immune based therapies targeted to intracranial tumors 9,10. Clinical trials have proven the safety and efficacy of FUS + MBs mediated opening of the BBB and/or BTB in human patients with Alzheimer's disease, amyotrophic lateral sclerosis (ALS), and glioblastoma. Furthermore, a clinical trial investigating this approach in the metastatic brain tumor setting is currently underway in patients with brain metastases of Her2+ breast cancer (NCT03714243) 11-13. Additionally, it is possible that FUS+MB BTB opening can modulate the immune microenvironment, which could augment or dampen the efficacy of immune-targeted therapies. In normal brain tissue, FUS + MB BBB opening has the potential to induce a sterile inflammatory response, with acute release of damage-associated molecular patterns (DAMPs), proinflammatory cytokines and chemokines, and trophic factors 14. This response was mostly resolved within 24 hours of treatment. Thus, it is possible that FUS+MB BTB opening could modulate the immune microenvironment to augment the efficacy of immune-targeted therapies in brain tumors.

Given the potential for FUS + MBs to be used along with immunotherapeutic agents, as well as the progression of FUS into clinical trials of patients with metastatic brain tumors, it is important to understand how FUS+MBs modulates the immune landscape of intracranial tumors. Here, using bulk RNA sequencing and flow cytometry, we investigated the effects of FUS+MBs BTB/BBB opening on several aspects of the melanoma brain tumor immune microenvironment, as well as effects in the meninges and draining lymph nodes.

## Results

### FUS+MBs Mediated BTB/BBB Opening in Intracranial B16F1cOVA Melanomas

**Figure 1:**
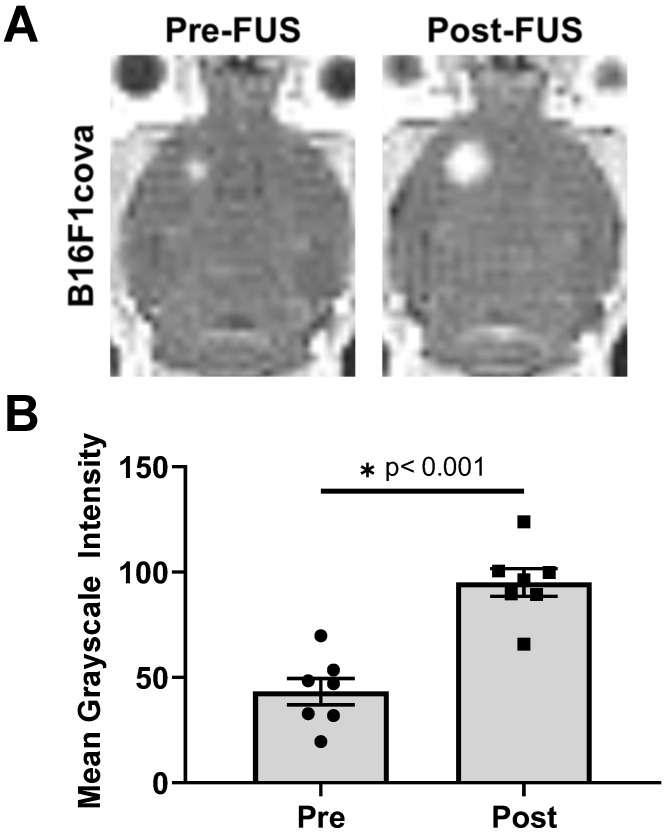
** Characterization of FUS + MBs-induced BTB/BBB opening by contrast enhanced MRI. A.** Representative images showing enhanced contrast Post-FUS**. B.** Quantification of pre- and post-FUS grayscale intensity for FUS+MBs treated tumors. Significance assessed by paired t test.

B16F1cOVA (B16F1 melanoma tumor cells that express the chicken ovalbumin antigen with the secretion sequence deleted) tumor cells (400,000 cells) were implanted into the striatum of wild-type mice. Three days later, FUS+MBs BTB/BBB disruption (1.1 MHz, 0.5 MPa, 0.5% duty cycle) was performed on a subset of tumor-bearing animals, with the remaining sham treated animals undergoing a single contrast enhanced MRI. The 0.5 MPa peak-negative pressure value was chosen to be well within the safe (i.e. stable cavitation) range at 1 MHz when used with this MB formulation in mice with brain tumors 15. For FUS+MBs treated animals, contrast enhanced MRI was performed prior to FUS+MBs treatment to visualize and target the tumor tissue and immediately following treatment to confirm BTB/BBB disruption, as shown in Figure 1A. The hyperintense region in the pre-FUS+MBs image corresponds to the brain tumor, whereas the larger hyperintense region in the post-FUS+MBs image qualitatively indicates enhanced gadolinium delivery, and thus successful BTB/BBB disruption. As a quantitative confirmation of BTB/BBB disruption, mean grayscale intensity for the tumor ROI was determined. Post-FUS+MBs grayscale intensity within the tumor ROI was significantly increased, indicating successful BTB/BBB disruption (Figure 1B).

### FUS+MBs Mediated BTB/BBB Opening in B16F1cOVA Tumors Elicits Increased Proinflammatory Gene Expression

**Figure 2:**
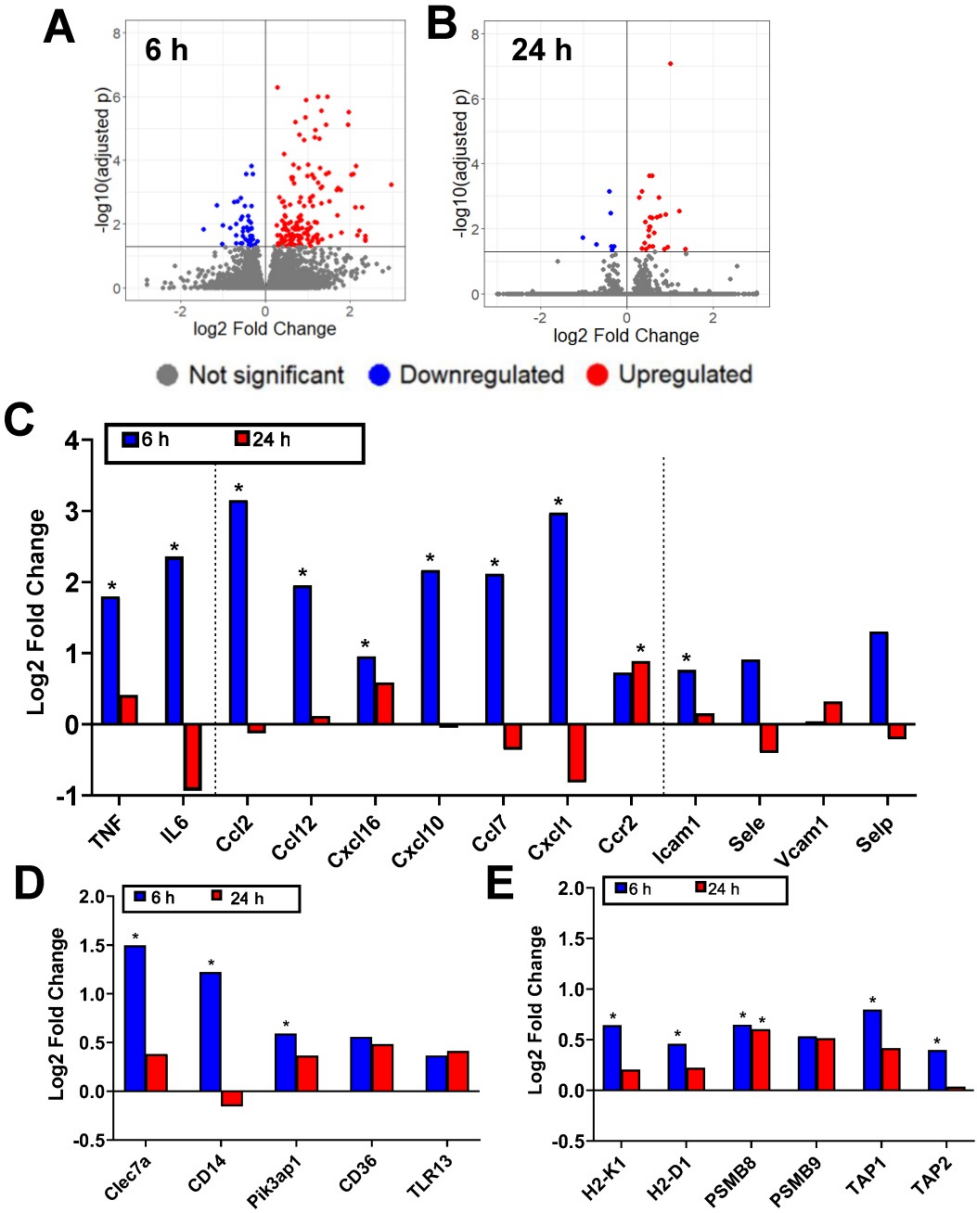
** Bulk RNA sequencing reveals that FUS+MBs mediated BBB/BTB opening elicits increased proinflammatory gene expression. A,B:** Volcano plots showing significantly upregulated and downregulated genes in FUS+MBs treated tumors compared to sham at (A) 6 and (B) 24 h post treatment.** C,D,E:** Log2 fold change of FUS+MBs treated vs. sham tumors at 6 hours and 24 hours post treatment. Data is displayed for selected mRNA transcripts related to** (C)** inflammatory cytokines, chemokines, and vascular cell adhesion molecules** (D)** pattern recognition receptors and signaling molecules and** (E)** MHC class I antigen presentation and processing. *adj p < 0.05. n = 3 for 24 h FUS+MBs group, n = 4 for all other groups.

FUS+MBs BTB/BBB disruption was performed on B16F1cOVA tumors 3 days following tumor cell implantation, using the protocol described above. FUS+MBs treated and sham tumors were harvested 6 and 24 hours later, and bulk RNA sequencing was performed on RNA isolated from tumor tissue. In FUS+MBs treated tumors, there were 203 transcripts that were significantly differentially expressed at the 6 hour time point and 37 transcripts at 24 hours (Figure 2A, B). Several transcripts related to inflammation were significantly upregulated at 6 hours post-FUS+MBs. Specifically, transcription of the proinflammatory cytokines tumor necrosis factor (*TNF*) and *IL-6* was significantly increased by roughly 3-fold and 5-fold, respectively (Figure 2C). Additionally, the transcripts for the chemokines *CCL2, CCL12*, *CXCL16*, *CXCL10*, *CCL7*, and *CXCL1* were all significantly increased 6 hours following FUS+MBs treatment (Figure 2C), while ICAM1 exhibited a significant 1.7-fold increase relative to sham animals at 6 hours (Figure 2C). Several transcripts related to pattern recognition receptors and signaling were also significantly differentially expressed. Transcripts of the pattern recognition receptors Clec7a and CD14 were increased by 2.8-fold and 2.3-fold, respectively, and the transcript for signaling adaptor molecule Pik3ap1 was increased by 1.5-fold (Figure 2D) 16-19. Lastly, a number of transcripts related to antigen processing and presentation were significantly differentially regulated. Expression of the classical MHCI molecules, H2-K1 and H2-D1, were increased by roughly 1.5-fold 6 hours following treatment (Figure 2E). PSMB8, a subunit of the immunoproteasome involved in antigen processing, was increased by about 1.5-fold at both 6 and 24 hours after treatment (Figure 2E) 20. Gene transcripts for TAP1 and TAP2, which are transporter proteins involved in shuttling peptides into the endoplasmic reticulum for loading onto MHCI molecules, were increased by 1.7 and 1.3-fold, respectively (Figure 2E) 21.

### FUS+MBs Mediated BTB/BBB Opening in B16F1cOVA Tumors Significantly Enriches Proinflammatory Gene Sets

**Figure 3:**
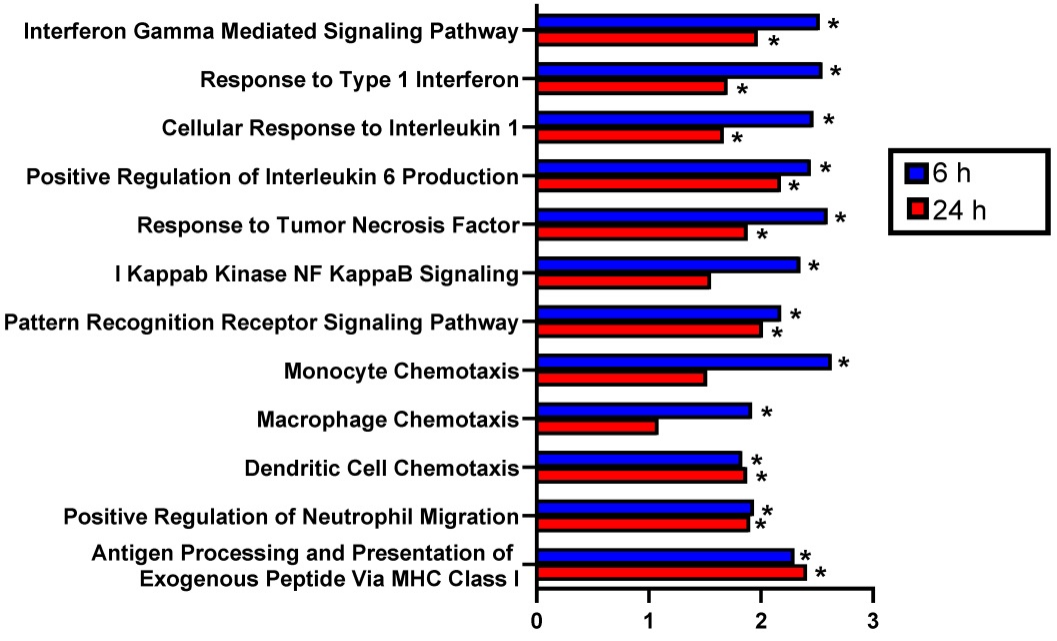
** Gene set enrichment analysis of RNA sequencing data indicates that FUS+MBs mediated BBB/BTB opening enriches proinflammatory gene sets.** Normalized enrichment scores for selected pathways of FUS+MBs treated relative to sham treated tumors at 6 and 24 h post FUS+MBs. *adj p < 0.05. n = 3 for 24 h FUS+MBs group, n = 4 for all other groups.

Beyond assessment of changes in individual transcripts, we performed gene set enrichment analysis to ascertain whether changes in gene expression were also associated with known biological processes**.** GSEA revealed numerous differentially expressed pathways related to inflammation in FUS+MBs treated tumors at 6 hours and 24 hours post-treatment. Specifically, normalized enrichment scores (NES) were significantly increased at both time points for several GO terms involving proinflammatory cytokines, including Interferon Gamma Mediated Signaling Pathway, Response to Type 1 Interferon, Cellular Response to Interleukin 1, Positive Regulation of Interleukin 6 Production, and Response to Tumor Necrosis Factor (Figure 3). Furthermore, the NES for Pattern Recognition Receptor Signaling Pathway was significantly increased at both time points, and I Kappab Kinase NF Kappab Signaling was increased 6 hours post-FUS (Figure 3). GO terms related to innate immune cell chemotaxis were enriched at both time points. Monocyte and Macrophage chemotaxis were increased at 6 hours, whereas Dendritic Cell Chemotaxis and Positive Regulation of Neutrophil Migration had significant NES at both time points (Figure 3). Lastly, Antigen Processing and Presentation of Exogenous Peptide via MHC class I was significantly enriched with FUS+MBs compared to sham treated tumors at both 6 and 24 hours (Figure 3). Based on these significantly enriched gene sets and transcript level data, FUS+MBs BTB/BBB disruption is generating an acute proinflammatory response in the tumor tissue. Since these proinflammatory processes may support the generation of an antitumor immune response, we chose to further investigate the impact of the inflammatory events stimulated by FUS+MBs BTB/BBB opening on the innate and adaptive cellular components of anti-tumor immunity.

### Flow Cytometry Analysis of Innate Immune Cell Representation in FUS+MBs Treated and Sham B16F1ZsGreenOVA Tumors

The increased expression of numerous proinflammatory cytokines, including IL6 and TNFα and chemokines involved in immune cell chemotaxis observed via bulk RNA sequencing, could support trafficking and maturation of various immune cell populations to and from the tumor tissue. Therefore, we tested whether myeloid and dendritic cell populations were enhanced within FUS+MBs treated tumors compared to sham at 2 and 4 days post treatment. We harvested treated and sham B16ZsGreenOVA tumors at the 2 and 4 day time points post treatment and examined innate immune cell phenotype and numbers via flow cytometry. For this flow cytometry experiment (see Figure S1 for the gating strategy), we used a different tumor model (B16F1ZsGreenOVA) to allow for tracking of the ZsGreen tumor antigen, as will be described in subsequent sections. No changes were observed in any of the evaluated cell types, which included DC (CD11c hi) and myeloid cells (CD11b+), as well as specific cellular subsets of DC (CD11c hi CD11b+ and CD11c hi CD103+) and other myeloid subsets (macrophages: CD11b+ F4/80+, monocytic myeloid cells: CD11b+ Ly6C hi, and granulocytic myeloid cells: CD11b+ Ly6C mid) in FUS+MBs treated tumors two days and four days post treatment (Figure S2). Thus, we observed no acute increase in the recruitment of innate immune cells despite enhanced chemokine expression.

### Dendritic Cell Activation Marker Expression in Intracranial B16F1ZsGreenOVA Tumors, Meninges, and Superficial Cervical Lymph Nodes

**Figure 4:**
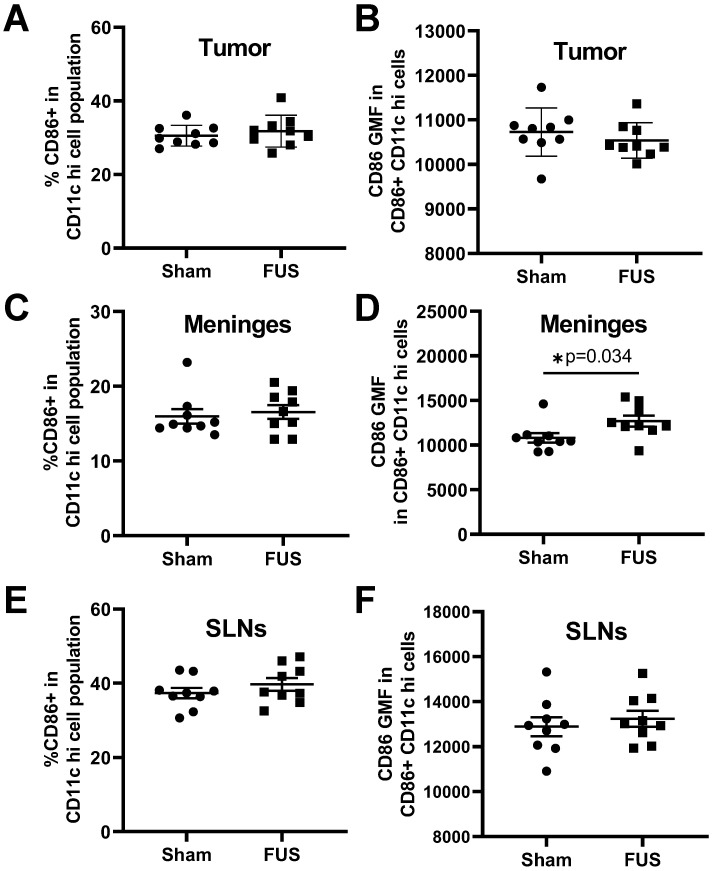
** Expression of the DC activation marker CD86 as assessed by flow cytometry 2 days post treatment. A,C,E:** Percentage of dendritic cells expressing CD86 in the (A) tumor (C) meninges and (E) superficial cervical lymph nodes of FUS+MBs treated and sham animals 2 d post FUS+MBs or sham treatment.** B,D,F:** Geometric mean fluorescence of CD86 in CD86 positive DCs in the (B) tumor (D) meninges and (F) superficial cervical lymph nodes of FUS treated and sham animals 2 d post FUS+MBs or sham treatment. Significance assessed by unpaired t tests.

Given the observed FUS+MBs induced gene signatures related to proinflammatory cytokine release and signaling, which can support dendritic cell maturation, we tested whether FUS+MBs treatment can increase DC maturity. As described above, FUS+MBs treated and sham B16F1ZsGreenOVA tumors were harvested, along with the meninges and superficial cervical lymph nodes (SLNs), which have been indicated to drain meningeal lymphatics 22 at 2 days and 4 days following treatment. We assessed DC expression of CD86, a costimulatory molecule that is increased upon DC maturation, to test whether FUS+MBs treatment induced increased DC maturity. For tumor-resident DCs, we found no changes in CD86 at either time point (Figure 4A,B; Figure S3A,B). In the meninges, we observed no changes in the proportion of DCs expressing CD86, but there was a significant increase in the amount of CD86 expressed in DCs two days post FUS+MBs (Figure 4C,D), with no changes observed at 4 days (Figure S3C,D). This could indicate increased meningeal DC maturity in the FUS+MBs treated animals 2 days post treatment. Additionally, we observed no significant changes in expression of CD86 in the DC population within the superficial cervical lymph nodes (Figure 4E,F, Figure S3E,F).

### Flow Cytometry Analysis of ZsGreen Antigen Uptake and OVA Peptide Presentation in DCs in the Tumor, Meninges, and SLN of Intracranial B16F1ZsGreenOVA Tumor-Bearing Mice

**Figure 5:**
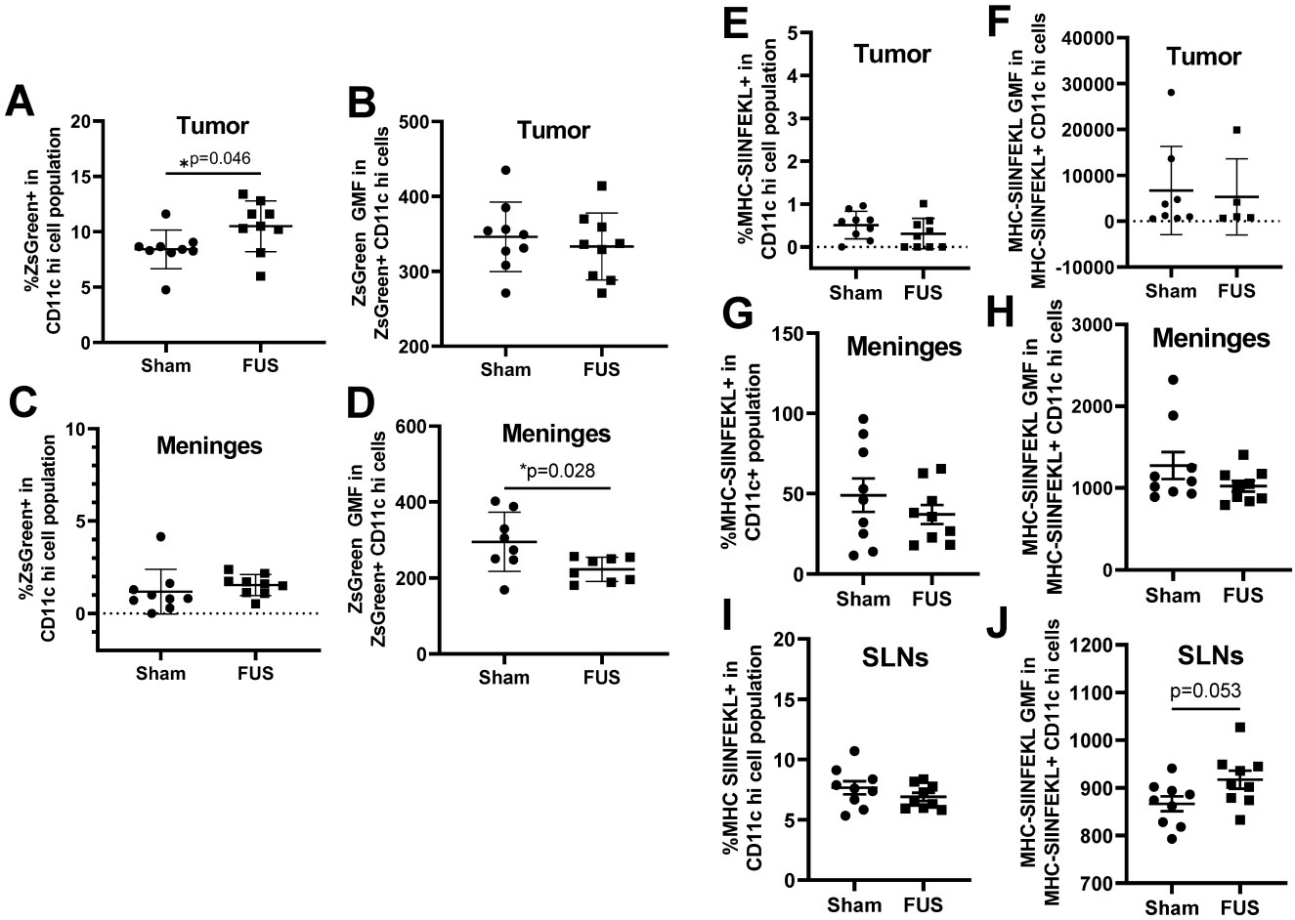
** ZsGreen antigen uptake and OVA peptide presentation by dendritic cells as assessed by flow cytometry 2 Days following treatment. A,C:** Percentage of ZsGreen positive dendritic cells in the (A) tumor and (C) meninges two days following treatment.** B,D:** Geometric mean fluorescence of ZsGreen in ZsGreen positive dendritic cells in the (B) tumor and (D) meninges two days following treatment. **E,G,I:** Percentage of MHC-SIINFEKL positive dendritic cells in the (E) tumor (G) meninges and (I) superficial cervical lymph nodes.** F,H,J:** Geometric mean fluorescence of MHC-SIINFEKL in MHC-SIINFEKL positive dendritic cells in the (F) tumor (H) meninges and (J) superficial cervical lymph nodes. Significance assessed by unpaired t tests.

Based on the gene expression signatures from bulk RNA sequencing, which indicated significant upregulation of several genes related to antigen processing and presentation, we tested whether FUS+MBs BTB/BBB opening altered dendritic cell antigen uptake and presentation. As previously mentioned, the tumor cells used in these studies were B16F1ZsGreenOVA cells, which are stably transfected to express MHC-restricted peptides derived from OVA in frame with ZsGreen fluorescent protein. This allowed us to use ZsGreen fluorescence as a marker for antigen uptake in the tumor (Figure S1F) and meningeal DCs. Additionally, we stained for the SIINFEKL peptide MHC class I (H-2K^b^) complex on DCs, indicating cross-presentation of the OVA257-364 peptide via MHC I. In tumor tissue, we detected an increased percentage of ZsGreen positive CD11c hi dendritic cells two days after FUS treatment, but no difference was seen in ZsGreen geometric mean fluorescence (GMF) in this population (Figure 5A, B). In the meninges, there were no changes in percentage of ZsGreen+ DCs, however there was a significant decrease in ZsGreen GMF two days post FUS (Figure 5C,D). This decrease in ZsGreen abundance may signify a downregulation of phagocytosis or degradation of acquired antigen within this cell population that occurs upon DC activation and maturation, which further supports increased meningeal DC maturity following FUS+MBs treatment, We saw no changes in MHC-SIINFEKL staining on DCs in the tumor or meninges (Figure 5E, F,G,H). There was also no change in the percentage of cells expressing the MHC-SIINFEKL complex in the superficial cervical lymph nodes 2 days post-FUS+MBs, but there was a trend towards increased GMF of MHC-SIINFEKL staining (Figure 5I,J). This may indicate increased antigen drainage to the lymph nodes. There were no significant changes in ZsGreen or MHC-SIINFEKL in DC populations 4 days post treatment (Figures S4).

### Endothelial Cell Adhesion Molecule Expression in Intracranial B16F1cOVA Tumors

**Figure 6:**
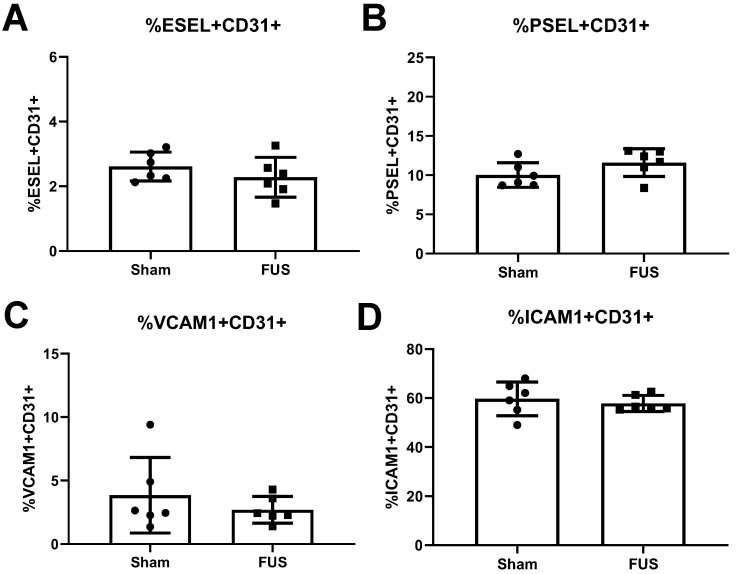
** Flow cytometry analysis of endothelial cell adhesion molecule expression on the vasculature 6 hours post-treatment. A,B,C,D:** Percentage of endothelial cells expressing (A) E-selectin (B) P-selectin (C) VCAM-1 and (D) ICAM-1 from FUS treated and sham B16F1cOVA tumors. Significance assessed by unpaired t tests.

The release of proinflammatory cytokines, such as TNFα, can induce expression of cell adhesion molecules on the surface of endothelial cells, ultimately contributing to leukocyte homing into inflamed tissue. Thus, we investigated whether FUS-induced inflammation led to differences in expression of cell adhesion molecules E-selectin, P-Selectin, ICAM-1, and VCAM-1 on intracranial B16F1cOVA tumor CD31+ endothelial cells 6 hours post FUS+MBs BTB/BBB opening. We saw no differences in the percentages of endothelial cells expressing these molecules in FUS+MBs treated tumors (Figure 6A-D). Additionally, we stained for E-selectin, ICAM-1, and VCAM-1 on tumor endothelial cells roughly 24 hours post FUS+MBs BTB/BBB opening in intracranial B16F1cOVA and in the less immunogenic B16F1 tumors. We saw no differences in expression of these cell adhesion molecules on B16F1cOVA tumors (Figure S5A-C), but found a significant decrease in the percentage of endothelial cells expressing E-selectin in FUS-treated B16F1 tumors (Figure S5D). However, we saw no changes in the other two cell adhesion molecules (Figure S5E,F). Thus, FUS+MBs induced inflammation in intracranial B16 melanoma tumors is insufficient to alter protein expression of cell adhesion molecules on endothelial cells at the tested time points.

### Activated T Cell Homing in Tumors and Meninges

**Figure 7:**
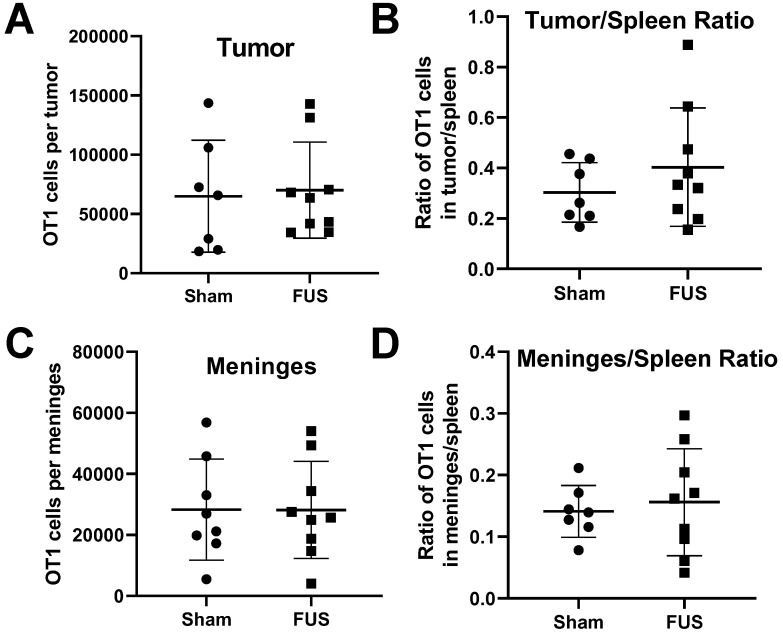
** Assessment of adoptively transferred activated T cell populations in the tumor and meninges by flow cytometry. A,C:** Number of transferred OT-1 T cells in the (A) tumor or (C) meninges of FUS+MBs treated or sham animals bearing intracranial B16F1cOVA tumors. **B,D:** Ratio of transferred OT-1 T cells in the (B) tumor or (D) meninges to the number in the spleen of FUS+MBs treated or sham animals bearing intracranial B16F1cOVA tumors. Significance assessed by unpaired t-tests.

Results from RNA sequencing showed increased expression of proinflammatory cytokines and chemokines in intracranial melanoma tumors 6 hours post-FUS+MBs treatment. We were interested in whether FUS+MBs treatment could enhance the ability of activated T cells to home to the tumor site, possibly through release of these soluble mediators. To test this, we performed an adoptive cell transfer of activated OT-1 cells (TCR transgenic CD8+ T cells specific for OVA) immediately prior to FUS+MBs BTB/BBB opening in B16F1cOVA tumors, 6 days after tumor implantation. Eighteen hours post treatment we harvested the tumors and meninges, and transferred cell numbers in these tissues were assessed by flow cytometry (see Figure S6 for gating strategy). There were no differences in the number of transferred OT-1 cells in the tumor or meninges in FUS+MBs treated versus sham animals (Figures 7A and C). Normalizing the number of transferred OT1 cells in the tumor and meninges to the number of OT1 cells in the spleen to account for possible variations in cell engraftment did not affect this result (Figure 7B and D). Furthermore, when activated T cells from Pmel TCR transgenic mice (whose TCR are specific for the gp100 antigen expressed by B16F1cOVA) were transferred 24 hours following FUS+MBs treatment of B16F1cOVA and harvested 5 hours later, we found no differences in the number of activated Pmel or non-specific T cells in the tumor or meninges (Figure S7).

## Discussion

We characterized the effect of FUS+MBs BTB/BBB opening in intracranial melanoma tumors on multiple facets of the immune landscape in the tumor, meninges, and CNS-draining lymph nodes. Bulk RNA sequencing of tumor tissue revealed increased expression of several proinflammatory cytokines and chemokines, as well as genes involved in pattern recognition receptor signaling and antigen processing and presentation via MHC class I in FUS+MBs treated tumors. We then utilized a similar B16F1 cell line expressing recombinant ZsGreen in frame with MHC class I and class II-restricted OVA peptides to assess FUS+MBs effects on innate immune cell numbers, DC expression of maturation markers, and DC tumor antigen uptake and presentation via flow cytometry. We observed increased CD86 expression and decreased ZsGreen antigen load in the meningeal DC population, and the percentage of ZsGreen-positive DCs in FUS+MBs treated tumors was increased two days post-treatment. Lastly, we tested whether FUS+MBs BTB/BBB opening could enhance expression of endothelial cell adhesion molecules or homing of subsequently administered activated T cells to the tumor site. We found no changes in expression of cell adhesion molecules on tumor endothelial cells and no changes in activated T cell homing in FUS+MBs treated tumors/meninges when cells were administered either immediately prior to or 24 hours following sonication. Thus, we observed that FUS+MBs mediated BTB/BBB opening of immunogenic intracranial B16F1cOVA tumors elicits some signatures of inflammation. Ultimately, however, the response is mild, transient, and unlikely to elicit a systemic response against the tumor without administration of immune adjuvants.

### Comparison of Response to MB Activation with FUS in Brain Tumors to Normal Brain Tissue

Despite marked differences between brain tumor tissue and normal brain tissue, proinflammatory signatures in FUS+MB treated tumors were consistent with sterile inflammatory responses (SIR) previously described in normal brain tissue 14,23. Since the BBB is meant to maintain homeostasis in the brain, BBB disruption by any means represents a significant perturbation of the local microenvironment. For instance, BBB opening alters the ionic balance in the tissue and facilitates extravasation of serum proteins, which can induce cellular stress responses and activation of microglia 24. Additionally, if applied with enough energy, FUS may induce mechanical damage in neurons and astrocytes, which may yield the release of DAMPs to initiate the SIR cascade. In contrast, within brain tumors, the BBB is already disrupted, cells are rapidly dividing and exposed to solid and mechanical stresses, and the microenvironment is characterized by tumor mediated immunosuppression. Despite these differences, we similarly observed a transient proinflammatory response in the tissue, characterized by increased proinflammatory cytokine, chemokine, and cell adhesion molecule transcripts 6 hours post-FUS+MBs, which included TNFα, IL-6, Ccl2, Ccl12, Cxcl10, Cxcl1, and ICAM-1. We did not, however, see increases in E-selectin or P-selectin at the RNA level, as previously seen in normal brain tissue. Additionally, gene set enrichment analysis of our RNA sequencing data provided evidence for increased pattern recognition receptor signaling (likely in response to DAMPs), interferon gamma mediated signaling, and NF-kappa B signaling, consistent with observations at the RNA and protein level in normal brain tissue 14.

Since we saw increased expression of several cytokines implicated in stimulation of cell adhesion molecule expression and observed increased ICAM-1 at the RNA level, we also assessed protein expression of cell adhesion molecules on tumor endothelial cells at 6 and 24 hours post-FUS+MBs BTB/BBB opening. We did not see differences in protein expression of E-selectin, P-selectin, ICAM-1, or VCAM-1 at 6 hours, or of E-selectin, ICAM-1, or VCAM-1 at 24 hours. While the previous study in normal brain tissue did not report protein data on E-selectin or VCAM-1, investigators reported a 4-fold increase in ICAM-1 protein 24 hours post FUS+MBs BBB opening, whereas here we saw no change in ICAM-1 protein expression 24 hours post FUS+MBs (despite detecting an elevation at the transcript level 6 hours post-FUS+MBs) 14. It is possible that even with increases in cytokines following FUS+MBs treatment, brain tumor endothelial cells do not respond to these stimuli in the same manner as normal vasculature. Furthermore, we observed higher expression of these cell adhesion molecules in B16F1cOVA tumors compared to contralateral brain tissue (data not shown), suggesting that the tumor endothelial cells may already be in a more activated state. Thus, further activation by FUS+MBs induced cytokines may not be possible, or the amount of cytokines released after FUS+MB treatment may be insufficient to raise the expression of adhesion molecules.

### Putative Antigen-Presenting Cell Response Mechanisms

FUS+MBs BTB/BBB opening induced increases in several chemokine transcripts involved in innate immune cell chemotaxis; however, we observed few changes in cell numbers in the tumor. Within the tumor site, we observed trends toward increased DC and other myeloid cell numbers 4 days post FUS+MBs, suggesting possible recruitment into the site of inflammation. Since we are only seeing snapshots of overall cell numbers at particular time points, more direct experiments would be needed to make definitive conclusions about FUS+MBs induced changes in myeloid cell trafficking.

DCs are professional antigen-presenting cells that act as a bridge between the innate and adaptive immune system; therefore, these cells are commonly recognized as the most important antigen-presenting cells in the generation of an antitumor immune response 25. In peripheral tissue, DCs exist in an immature form in which they primarily endocytose materials and accumulate antigens. Immature DCs are poor antigen presenters and may even contribute to tolerance. Molecules such as PAMPs and DAMPs activate pattern recognition receptors on DCs, providing a maturation stimulus. Additionally, upon maturation, DCs downregulate endocytosis, activate machinery involved in antigen processing and presentation and thus express higher levels of MHC-peptide complexes, and increase expression of T cell co-stimulatory molecules, making them effective T cell activators 25. Here, we observed enhanced gene expression signatures related to pattern recognition receptor signaling and antigen processing and presentation at both 6 and 24 hours following FUS+MBs treatment via bulk RNA sequencing. The bulk sequencing approach does not allow us to attribute these changes to specific cell populations within the tissue, so it is possible that these changes could be occurring in any infiltrating cell type including microglia, DCs, or the tumor cells themselves. Due to the importance of these processes within the DC population for the initiation of an antitumor immune response, we chose to interrogate several aspects of the DC response in the tumor, as well as in the meninges and tumor draining lymph nodes via flow cytometry. Though further experiments would be needed to examine whether gene expression signatures in the meninges and lymph nodes mirror those seen in the tumor, it is possible that these sites are exposed to soluble factors released in the tumor upon FUS+MBs treatment and may also exhibit responses in the DC population. Furthermore, upon maturation, DCs are known to migrate to draining lymph nodes, thus it is possible that DCs from the tumor site are trafficking to the meninges and SLNs, which could manifest as changes in the DC populations of these tissues.

Since stimulation of pattern recognition receptors via DAMPs and inflammatory cytokines such as TNFα can stimulate DC maturation, we assessed expression of the DC maturation marker CD86, a T cell costimulatory molecule that is upregulated upon maturation. Two days after treatment, the meningeal DC population expressed significantly higher levels of CD86. It is unclear why CD86 expression was increased in the meninges, but not in the tumor or draining lymph nodes of FUS+MBs treated animals. As mentioned previously, DC maturation can prompt DC migration to the lymph nodes, so it is possible that once DCs in the tumor receive the maturation stimulus they migrate away from the tumor site, thus we are unable detect changes in maturation within the tumor DC population. This could be the source of the DCs expressing increased levels of CD86 in the meninges. However, we did not detect increased DC maturity in the draining lymph nodes of FUS+MBs treated animals, as would be expected if mature DCs were trafficking to the nodes. Alternatively, meningeal DCs may be more exposed to cytokines and DAMPs. To further interrogate these possibilities, we would need to perform experiments dedicated to studying DC trafficking in response to FUS+MBs BTB/BBB opening.

Additionally, we looked at ZsGreen uptake and MHC-SIINFEKL expression to assess antigen acquisition and presentation. Along with increased CD86 expression, the DCs isolated from the meninges showed lower ZsGreen fluorescence. This could be due to degradation of the antigen and indicative of a switch from an immature phagocytic phenotype to a more mature phenotype for lymphocyte priming 25. The increased percentage of ZsGreen-positive DCs in the tumors of FUS+MBs treated mice suggests increased antigen uptake within the tumor following FUS+MBs BTB/BBB opening. Factors such as increased antigen from cell death, increased phagocytosis, or enhanced distribution of antigen throughout the tumor from FUS+MBs induced changes in interstitial transport could contribute to this finding 15. Staining for the MHC-SIINFEKL complex on DCs revealed a trend towards increased abundance within the superficial cervical lymph nodes of FUS+MBs treated animals. If DCs within the tumor have increased processing and presentation of peptide antigen via MHC class I, they could then traffic to the lymph nodes where they can display MHC-SIINFEKL complexes. Alternatively, increased drainage of tumor antigen to the superficial cervical lymph nodes in FUS+MB treated tumors could increase presentation of MHC-SIINFEKL on DCs in the SLNs.

### MHC Class I Molecule Response and Mechanisms

RNA sequencing revealed, at 6 hours post FUS+MBs, the increased expression of both classical MHC I molecules, H2-K1 and H2-D1. In fact, H2-K1, the MHC class I molecule that presents the OVA257-264 peptide, was the most significantly differentially expressed gene in FUS+MBs treated tumors at the 6 hour time point. As discussed previously, this could be due to increased expression on antigen-presenting cells such as DCs, which would be beneficial for priming of an antitumor immune response. However, it is also possible that the tumor cells are upregulating expression of class I MHC molecules. Furthermore, the increased gene signatures related to antigen processing and presentation could be occurring in the tumor cells, as opposed to antigen-presenting cells. This fits with the fact that we did not see increased expression of the MHC-SIINFEKL complex on DCs within the tumor or meninges. Future studies should determine whether this response is occurring in tumor cells, as this could also be beneficial to anti-tumor immunity by making the tumor cells more visible to tumor specific effector T cells. It is known that downregulation of MHC I expression is one mechanism used by tumor cells to evade detection by the immune system 26-29. Expression of MHC I and genes involved in antigen processing and presentation have been shown to be increased in response to type I and type II interferons 20,30. Though we did not observe an increase of IFN gamma, alpha, or beta at the transcript level, gene set enrichment indicated increased interferon gamma and type I interferon signaling. Thus, it is possible that an acute release of interferons led to increased MHC I expression and expression of genes related to antigen processing and presentation in the tumor and/or tumor-infiltrating immune cell populations 20,30.

### T Cell Responses to FUS+MBs Mediated BTB/BBB Opening

The relatively early time points chosen here to evaluate tissue responses to FUS+MBs treatments are more conducive to detecting changes in the innate component of the anti-tumor immune response rather than adaptive T cell responses, since de novo T cell responses take longer to manifest. Given the limited and transient nature of the proinflammatory response generated by FUS+MBs, we hypothesize that we would not detect FUS+MBs induced increases in T cell activation. While we did not assess de novo T cell response, given the putative increase in chemokine expression and adhesion molecules, we did investigate the ability of FUS+MBs BTB/BBB opening to augment homing of adoptively transferred activated T cells to intracranial melanoma tumors. Previous studies have demonstrated that greater accumulation of HER-2 specific NK-92 cells in a model of HER2+ breast cancer brain metastasis was achieved when cells were administered immediately prior to FUS+MBs BTB/BBB disruption 31. For this reason, we administered activated T cells immediately prior to FUS treatment. An additional 24 hour post-FUS time point was tested as well. Nonetheless, we found no differences in T cell homing to tumors with FUS+MBs BTB/BBB opening.

## Conclusions

We used RNA sequencing and flow cytometry to assess acute immune responses to FUS+MBs BTB/BBB opening in intracranial melanoma tumors. This response was characterized by enhanced gene signatures related to proinflammatory cytokines, chemokines, pattern recognition receptor signaling and antigen processing and presentation. Additionally, the flow cytometry data supports the notion that FUS+MBs can increase antigen presence or distribution within the tumor and contribute to DC maturation. Overall, the observed response to FUS+MB BTB/BBB opening in intracranial melanoma tumors is mild and transient; however, we submit that understanding this response will be important for the rational design of FUS+MBs mediated therapeutic delivery approaches.

## Materials and Methods

### Animals

Wild-type male C57BL6 mice were purchased from Charles River or NCI at 8-10 weeks of age. Male CD45.1 mice for adoptive transfer experiments and the 6 hour homing receptor experiment were purchased at 8-10 weeks of age from NCI. OT1 mice were initially purchased from Jackson labs and maintained at the University of Virginia. Pmel mice were purchased from Jackson. All animal experiments were approved by the Animal Care and Use Committee at the University of Virginia and conformed to the National Institutes of Health regulations for the use of animals in research.

### Tumor Implantation

Mice were anesthetized with a mixture of ketamine (40 mg/kg; Zoetis, Kalamazoo, MI) and Dexdomitor (0.2 mg/kg, Zoetis, Kalamazoo, MI) in 0.9% sterile saline. Mouse heads were depilated and buprenorphine was administered subcutaneously. Mice were then placed on a stereotaxic frame to position and secure the heads. The surgical site was prepared with alternating scrubs of alcohol and iodine and a midline scalp incision was made. A burr hole was drilled 2 mm to the right and 1 mm anterior to the intersection of the bregma and midline of the skull, to target the striatum as the injection site. B16F1, B16F1cOVA, or B16F1ZsGreenOVA (obtained from the Krummel lab at the University of California San Francisco 32) cells were loaded into a 10 μl Hamilton syringe mounted on the stereotaxic frame. The syringe was then placed in the burr hole and lowered to a depth of 4 mm below the skull and withdrawn 1 mm, for a final depth of 3 mm below the skull surface. A total volume of 2 μl of cell suspension (either 1e5, or 4e5 cells total) was injected over the course of 4 minutes. Almost all experiments utilized a cell implantation of 4e5 tumor cells, however, since B16F1 tumors were found to grow faster than B16F1cOVA tumors, 1e5 B16F1 cells were implanted for the endothelial cell adhesion molecule experiment. After one additional minute, the needle was slowly removed from the brain. Mice were sutured and moved to a heating pad for recovery. Anesthesia was reversed with Antisedan.

### BTB/BBB Opening with MR Image-Guided FUS and Microbubbles

Depending on experimental design and cell number implanted, FUS+MBs treatments were applied at either day 3 or day 6 after tumor cell implantation. Mice were anesthetized with a mixture of Ketamine (40 mg/kg; Zoetis, Kalamazoo, MI) and Dexdomitor (0.2 mg/kg, Zoetis, Kalamazoo, MI) in 0.9% sterile saline and tail veins were cannulated to allow for intravenous injections. The MR-guided FUS system (RK-100, FUS Instruments) sat directly on the patient table of a clinical 1.5T MRI scanner (Siemens Avanto). Mice were placed supine on the MR-guided FUS system with the skull sonically coupled to a 1.1 MHz spherically focused ultrasound transducer (with a 550 kHz hydrophone mounted in the center for passive cavitation detection) immersed in a degassed water bath. For the general treatment procedure, MultiHance gadolinium contrast agent (Bracco Diagnostics) was administered intravenously and a pre-FUS contrast-enhanced MR image of the entire brain was acquired using a custom-built 3-cm loop receive RF coil and three-dimensional spoiled gradient echo pulse sequence. Pulse-sequence parameters for all images were as follows: TR/TE = 11/5.33 ms, flip angle = 15°, readout bandwidth = 250 Hz/Px, FOV = 46×67×45 mm, resolution = 0.35 mm isotropic, total time per image = 3:05.

A sonication pattern was chosen based on the tumor size at the time of treatment. Either a single target spot (day 3 treatment) or a grid of 4 spots (100,000 or 400,000 cells, day 6 treatment) were chosen from this pre-sonication MR image to cover the entire tumor and surrounding tissue. To open the BTB/BBB, albumin-shelled MBs (1 x 10^5^/gram body weight; manufactured as previously described 33-35) were intravenously injected and FUS was applied to the targets using a specified peak negative pressure (0.5 MPa measured in water). FUS was applied in 10 ms pulses with a 2 s pulsing interval (i.e. 0.5% duty cycle) for a total of 2 minutes. Animals were then re-injected with gadolinium contrast agent and post-sonication contrast-enhanced MR images were acquired to confirm BTB/BBB opening. Once the treatment was completed, mice were given Antisedan to reverse the anesthesia and allowed to recover on a heating pad. Sham animals received only a single Gadolinium-enhanced MR imaging session. For the 24 hour homing receptor and adoptive T cell transfer experiments, animals in the no FUS group were given a single injection of MBs following the MRI.

### Grayscale Intensity Analysis

For each FUS+MBs treated animal, a region of interest (ROI) was drawn to encompass the hyperintense region in the pre-FUS image using ImageJ. This ROI was used to quantify mean grayscale intensity in the pre-FUS+MBs and post-FUS+MBs image of that animal. The background mean grayscale intensity for the pre-FUS+MBs and post-FUS+MBs image was quantified by placing the ROI on the contralateral side of the brain. These background measurements were subtracted from the tumor ROI measurements to obtain the reported pre and post-FUS values.

### RNA Sequencing

For RNA sequencing, 400,000 B16F1cOVA cells were implanted into the brain as previously described, 3 days prior to FUS+MBs treatment. For FUS+MBs treatment, a single sonication spot was applied in the presence of circulating microbubbles at the tumor location. Tumor tissue was harvested 6 hours and 24 hours following FUS+MBs application for treated and sham mice. Immediately following euthanasia, sham and FUS+MBs treated tumors were excised, placed in RNAlater (Qiagen) and stored at -80°C. RNA extraction was performed using the RNeasy Mini Kit (Qiagen). mRNA was isolated using the NEBNext Poly(A) mRNA Magnetic Isolation Module (New England Biolabs, Ipswich, Massachusetts) followed by library preparation using the NEBNext Ultra II Directional RNA Library Prep Kit for Illumina (New England Biolabs). Sequencing was performed using a NextSeq 500 (Illumina, San Diego, California) at a target depth of 25 million 2 x 75 bp paired end reads per sample. Reads were quasi-mapped to the mouse genome (mm10 assembly, modified to include the cOVA transgene) and quantified at the transcript level using Salmon v0.11.2 followed by summary to the gene level using tximport v1.10.1 36,37. Differential gene expression was performed with DESeq2 v1.22.2 38. Gene set enrichment analysis was performed with the MSigDB canonical pathways gene sets using FGSEA v1.8.0 run with 100,000 permutations 39,40.

### Flow Cytometry Analysis of Post-FUS+MBs Immune Cell Infiltration

Tumors, meninges, superficial lymph nodes, and spleen were harvested at day 5-8 after tumor injection. 3 minutes prior to harvest mice were injected with anti-CD45 to identify cells that remained in the vasculature. Tumors, lymph nodes, and spleen were homogenized and filtered through 70 µm mesh. Meninges were separated from the skull cap and filtered through 70 µm mesh. Spleens were treated with RBC lysis (Sigma). Samples were stained for viability with Aqua, incubated with CD16/32 antibody and 2% normal mouse serum to block Fc receptors, and then stained with dextramer (in T cell panels). Staining was done in FACS buffer (PBS containing 2% BSA, 0.08% sodium azide). Next, samples were stained with a variety of surface markers (see below) and then preserved using FACS lysis (BD). Samples were acquired on an Attune NxT flow cytometer and the data was analyzed using FlowJo (TreeStar) and Prism (Graphpad) software. The staining panel was as follows: Aqua Live/dead (Life Technologies); CD16/32 (clone 90; eBioscience), CD44 (clone IM7; eBioscience), CD45 (clone 30-F11; eBioscience), CD45.1 (clone A20; eBioscience), CD45.2 (clone 104; eBioscience), CD103 (clone 2E7; eBioscience), MHC SIINFEKL (clone 25-D1.16; eBioscience), Thy1.1 (clone HIS51; eBioscience); CD8 (clone 53-6.7; eBioscience), CD11b (clone M1/70; BioLegend), CD11c (clone N418; BioLegend), CD86 (clone GL-1; BioLegend), F4/80 (clone BM8; BioLegend), Ly6C (clone HK1.4; BioLegend).

### Flow Cytometry Analysis of Endothelial Cell Adhesion Molecule Expression

Tumors were harvested 6 or 24 hours following FUS+MBs BTB/BBB opening. The tumors were minced and placed in digestion media containing 0.42U/ml Liberase TM (Roche). Samples were digested at 37 °C for 15 minutes and triturated every 5 minutes. Samples were homogenized (using glass homogenizers) and filtered through 70 µm filters. Subsequently, all samples were centrifuged at 1500 rpm for 15 minutes. The pellets were resuspended in CD45+ magnetic beads (Miltenyi Biotech) with Fc Block (1:1000) and incubated for 15 minutes at 4 °C. Samples were washed with AwesomeMacs Buffer and centrifuged at 1500 rpm for 5 minutes. Samples were separated with autoMACS Pro Separator with POSSEL AutoMACS protocol. The CD45 negative fraction was pelleted and stained with CD31 endothelial cell panel. Cells were Fc blocked and stained with fluorescent antibodies for CD31 (clone 390, eBioscience), CD45 (clone 30-F11, Biolegend), E-sel (clone 10E9.6, DB), P-selectin (clone RB40.34, DB), ICAM-1 (clone YNI/1.7.4, Biolegend), VCAM-1 (clone 429, BD) and Live/dead Aqua stain (eBioscience). Cells were fixed in 2% PFA.

### Activated T Cell Adoptive Transfer

Activated OT1 or Pmel T cells were generated by transferring 10,000 OT1 or Pmel T cells i.v. into naïve mice and priming with CD40 agonistic antibody (100 ug, clone FGK45, BioXcell), poly I:C (75 ug, Invivogen), and ovalbumin (500 μg, Sigma) or gp100 25-33 peptide (200 ug, GenScript). Seven days later CD8 T cells were magnetically enriched (eBioscience 8804-6822-74) from spleens. Approximately 1 million CD8 T cells were transferred i.v. following FUS + MB BTB/BBB opening. Activated wild-type T cells were generated by culturing splenocytes for three days *in vitro* with agonistic antibodies for CD3 (5 ug/mL, eBioscience) and CD28 (2 ug/mL, eBioscience) plus IL-2 (10 IU/mL). Approximately 3 million CD8 T cells were transferred i.v. following FUS + MB BTB/BBB opening. Tumors, spleens and meninges were harvested 5 or 24 hours after cell transfer. CD45 antibody was injected i.v. 3 minutes prior to harvest to label circulating cells. This allowed us to separate the extravascular cells (no labeling with CD45 antibody) of interest from those in blood vessels (labeled with CD45 antibody). Transferred OT1 cells were identified by Thy mismatch (i.e. OT1 cells from Thy1.1 donor into Thy1.2 host). Pmel cells were identified by Thy or CD45 mismatch (i.e. Pmel cells from Thy1.1 CD45.2 donor into Thy1.2 CD45.1 hosts).

## Supplementary Material

Supplementary figures.
