# Supplementary material for: Immunomodulation of intracranial melanoma in response to blood-tumor barrier opening with focused ultrasound
Source: Theranostics. 2020 Jul 11;10(19):8821–33. doi: 10.7150/thno.47983 (PMC7392000; doi:10.7150/thno.47983)
Supplement: Supplementary file 1 — Supplementary figures. [file thnov10p8821s1.pdf]

Supplementary Figures

Immunomodulation of intracranial melanoma in response to blood-tumor barrier opening with focused ultrasound

Colleen T. Curley[1], Aaron D. Stevens[2], Alexander S. Mathew[1], Katarzyna Stasiak[3], William J. Garrison[1], G. Wilson Miller[1,4], Natasha D. Sheybani[1,*], Victor H. Engelhard[3], Timothy N.J. Bullock[2,*], and Richard J. Price[1,4,*]

[1]Department of Biomedical Engineering, University of Virginia, Charlottesville, VA
[2]Department of Pathology, University of Virginia, Charlottesville, VA
[3]Carter Immunology Center and Department of Microbiology, Immunology, and Cancer Biology, Charlottesville, VA
[4]Department of Radiology & Medical Imaging, University of Virginia, Charlottesville, VA

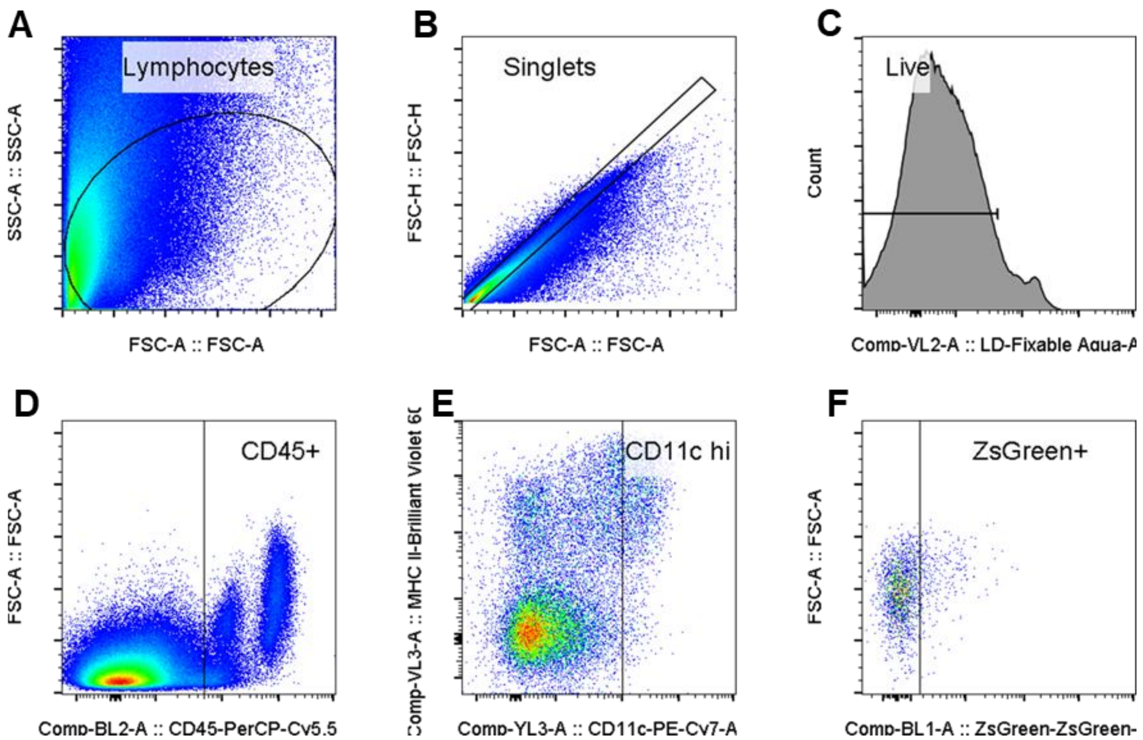

Figure S1. Gating strategy for identifying DCs and ZsGreen^{+} DCs. A: Scatter. **B**: Singlets. **C**: Live cells. **D**: CD45^{+}. **E**: CD11c^{hi} DCs. **F**: ZsGreen^{+}.

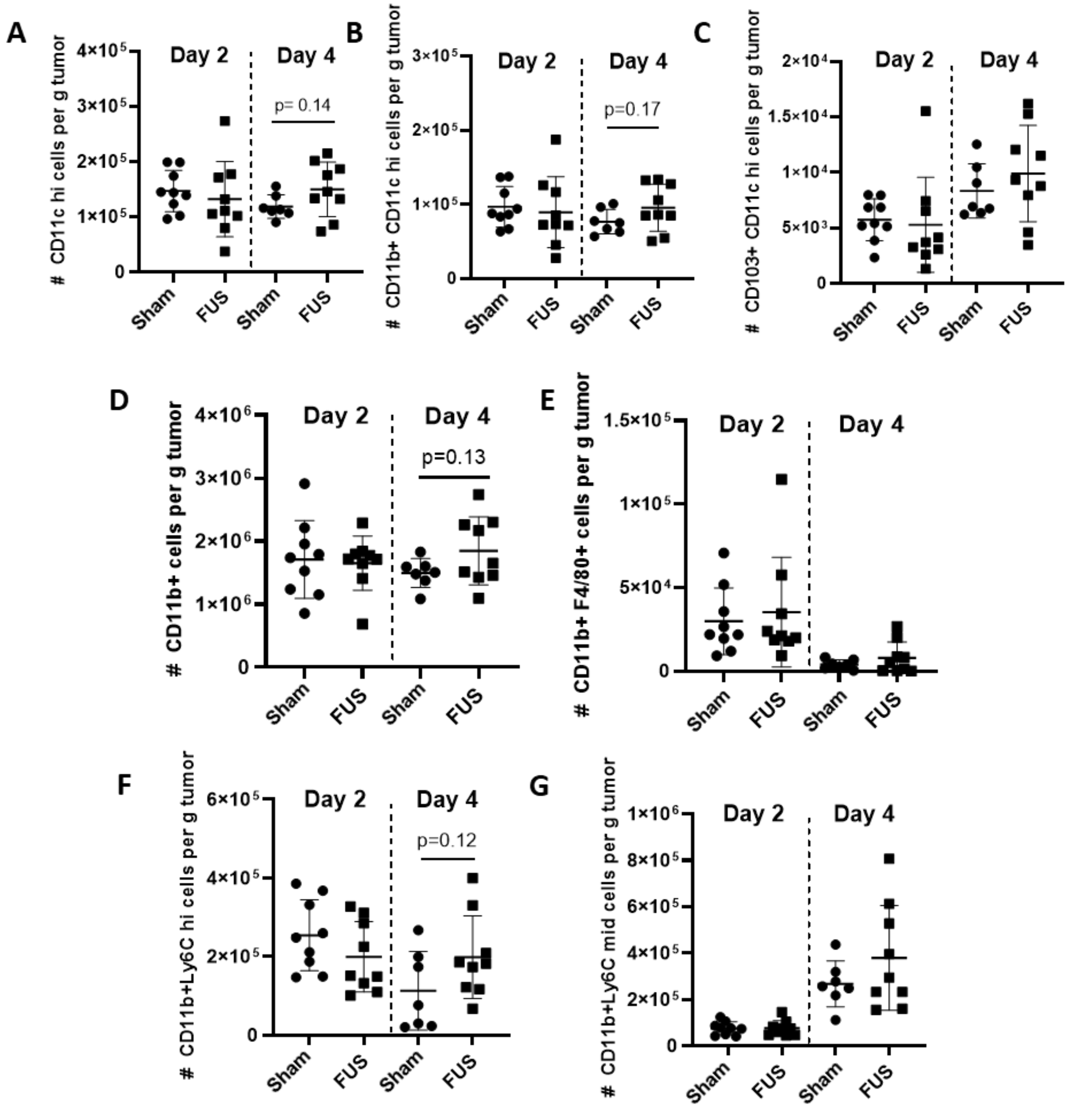

Figure S2. Innate immune cell populations in FUS+MBs treated and Sham control tumors. A,B,C: Number of (A) dendritic cells and (B,C) dendritic cell subsets per gram of tumor two and four days post treatment assessed by (A) CD11c hi (B) CD11c hi CD11b+ (C) CD11c hi CD103+. **D,E,F,G:** Number of (D) myeloid cells and (E,F,G) myeloid cell subsets per gram of tumor two and four days post treatment assessed by (D) CD11b+ (E) CD11b+ F4/80+ (F) CD11b+ Ly6C hi (G) CD11b+ Ly6C mid. Significance assessed by unpaired t tests between Sham and FUS+MBs treated tumors at each time point.

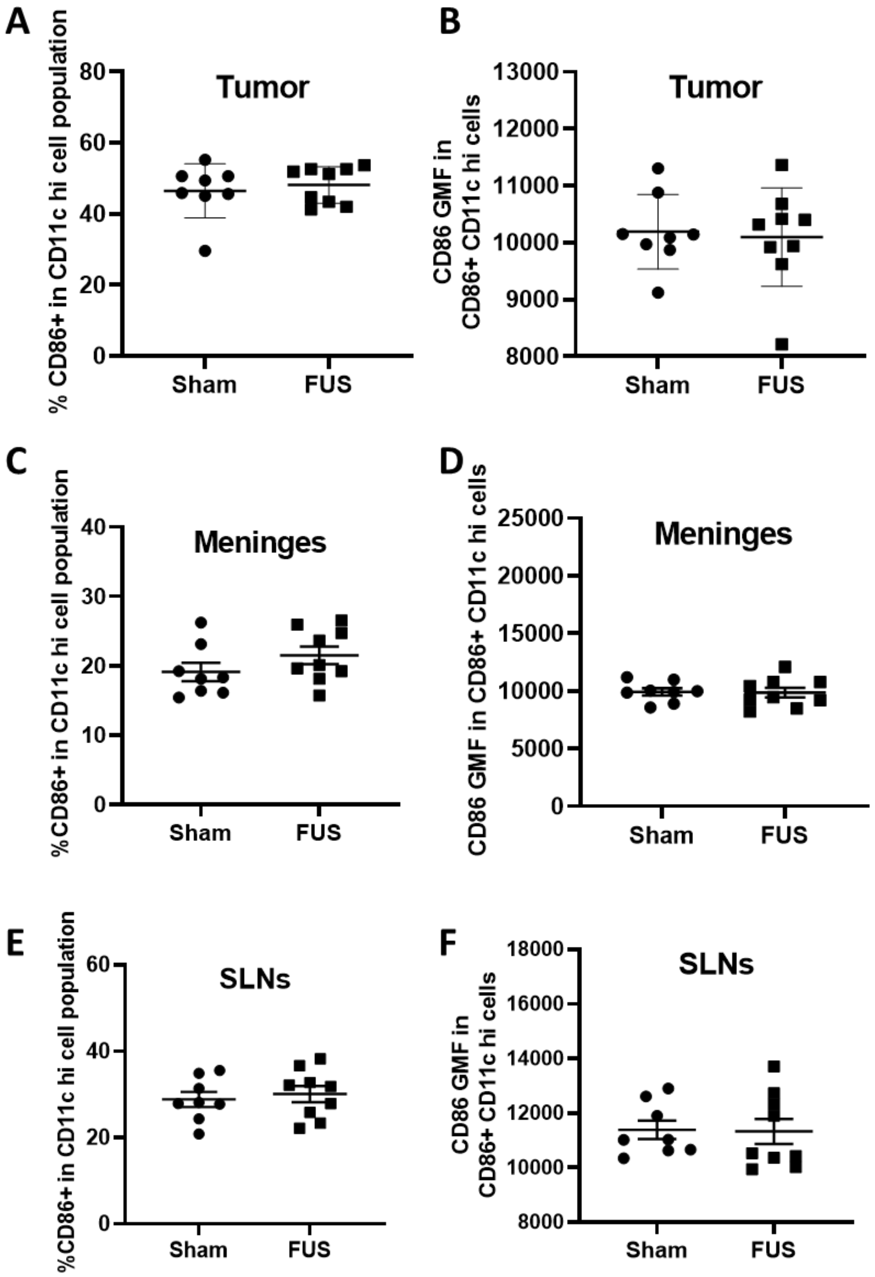

Figure S3. Expression of DC activation markers in FUS+MBs treated and Sham control tumors 4 days post treatment. A,C,E: Percentage of dendritic cells expressing CD86 in the (A) tumor (C) meninges and (E) superficial cervical lymph nodes of FUS+MBs treated and Sham animals 4 days post treatment. **B,D,F:** Geometric mean fluorescence of CD86 in CD86 positive DCs in the (B) tumor (D) meninges and (F) superficial cervical lymph nodes of FUS+MBs treated and Sham animals 4 days post treatment. Significance assessed by unpaired t tests between Sham and FUS+MBs treated animals.

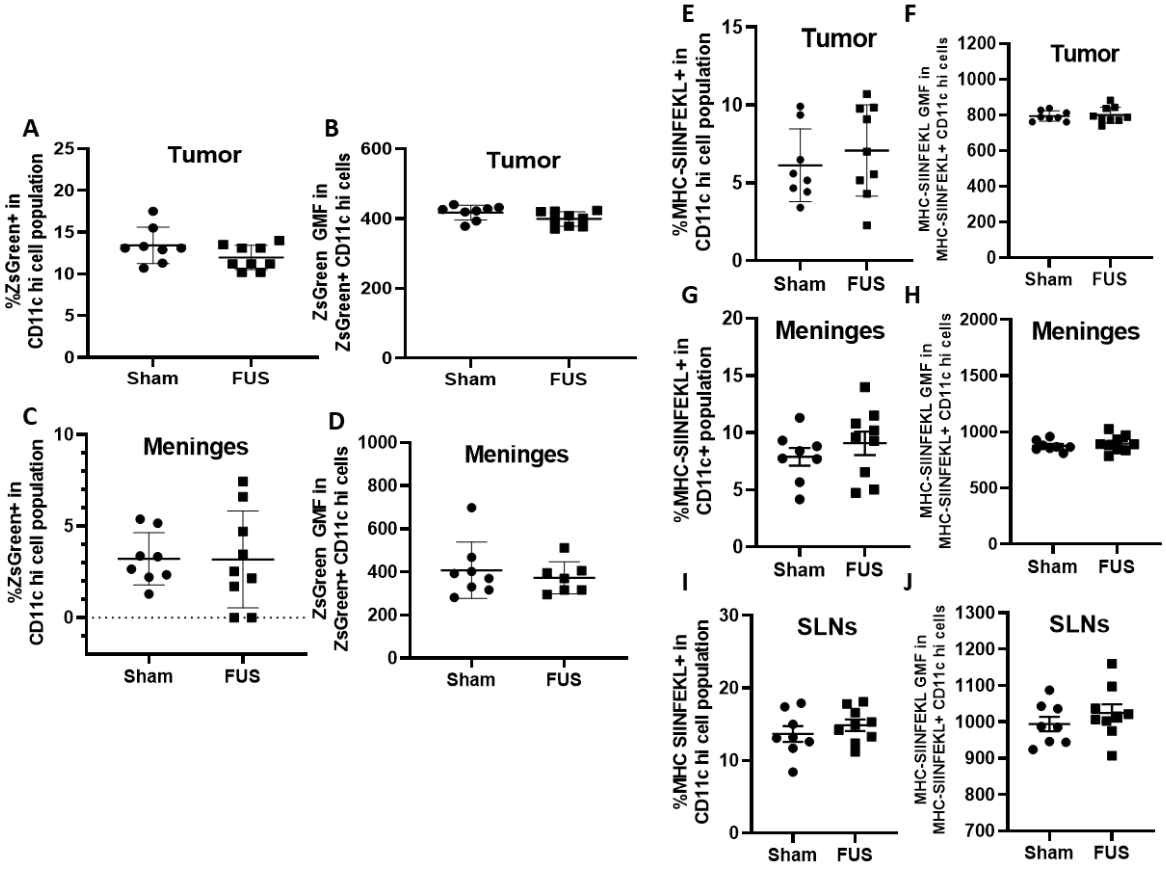

Figure S4. Antigen uptake and presentation by dendritic cells 4 days following FUS+MBs treatment. A,C: Percentage of ZsGreen positive dendritic cells in the (A) tumor and (C) meninges 4 days following treatment. **B,D:** Geometric mean fluorescence of ZsGreen in ZsGreen positive dendritic cells in the (B) tumor and (D) meninges 4 days following treatment. **E,G,I:** Percentage of MHC-SIINFEKL positive dendritic cells in the (E) tumor (G) meninges and (I) superficial cervical lymph nodes. **F,H,J:** Geometric mean fluorescence of MHC-SIINFEKL in MHC-SIINFEKL positive dendritic cells in the (F) tumor (H) meninges and (J) superficial cervical lymph nodes.

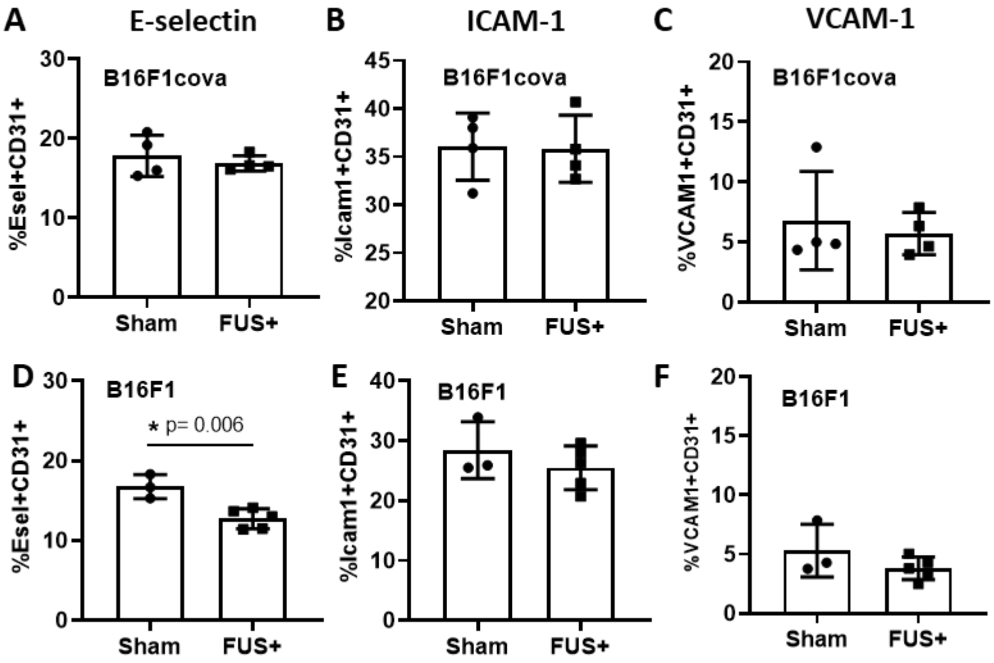

Figure S5. Endothelial cell adhesion molecule expression on the vasculature of FUS+MBs treated and Sham tumors 24 h post-treatment. A,B,C: Percentage of endothelial cells expressing (A) E-selectin (B) ICAM-1 or (C) VCAM-1 from FUS+MBs treated and Sham B16F1cOVA tumors. **D,E,F:** Percentage of endothelial cells expressing (D) E-selectin (E) ICAM-1 or (F) VCAM-1 from FUS treated and Sham B16F1 tumors. Significance assessed by unpaired t tests.

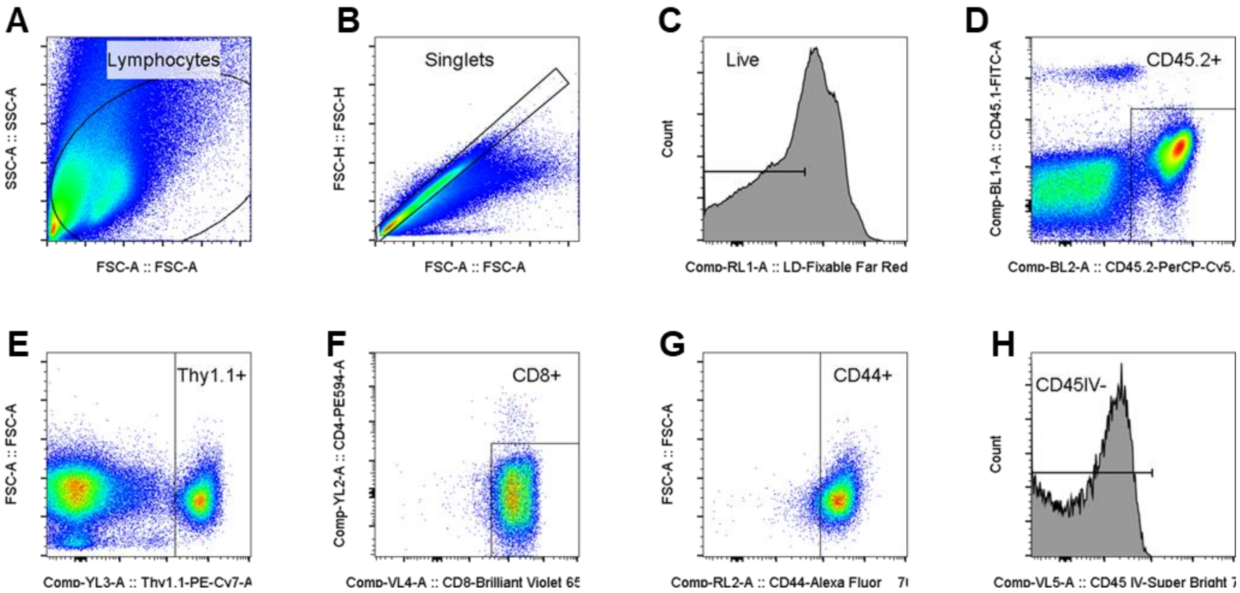

Figure S6. Gating strategy for identifying OT1 T cells. A: Scatter. **B**: Singlets. **C**: Live cells. **D**: CD45.2$^+$. **E**: Thy1.1$^+$. **F**: CD8$^+$. **G**: CD44$^+$. **H**: Extravascular cells (i.e. negative for i.v. injected CD45 antibody).

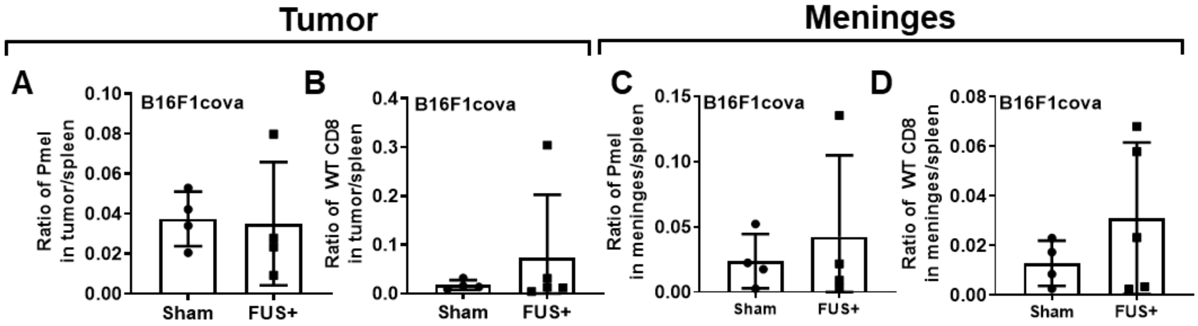

Figure S7. Adoptively transferred activated T cell populations in the tumor and meninges of FUS+MBs treated and Sham animals. A,C: Ratio of transferred Pmel T cells in the (A) tumor or (C) meninges to the number in the spleen of FUS+MBs treated or Sham animals bearing intracranial B16F1cOVA tumors. **B,D:** Ratio of transferred non-specific T cells in the (B) tumor or (D) meninges to the number in the spleen of FUS treated or Sham animals bearing intracranial B16F1cOVA tumors.
